# Ultrasonic Waves Regulate Antioxidant Defense and Gluconeogenesis to Improve Germination From Naturally Aged Soybean Seeds

**DOI:** 10.3389/fpls.2022.833858

**Published:** 2022-03-28

**Authors:** Yutao Huang, Gaofu Mei, Xujun Fu, Yang Wang, Xiaoli Ruan, Dongdong Cao

**Affiliations:** ^1^Institute of Crop and Nuclear Technology Utilization, Zhejiang Academy of Agricultural Sciences, Hangzhou, China; ^2^The Key Laboratory of Quality Improvement of Agricultural Products of Zhejiang Province, College of Advanced Agricultural Science, Zhejiang Agriculture and Forestry University, Hangzhou, China; ^3^Zhejiang Nongke Seed Co. Ltd., Hangzhou, China

**Keywords:** antioxidant defense, aged seeds, gluconeogenesis, seed germination, soybean, ultrasonic waves

## Abstract

Soybean seeds contain substantial triacylglycerols and fatty acids that are prone to oxidation during storage, contributing to the dramatic deterioration of seed vigor. This study reports an ultrasonic waves treatment (UWT), which is a physical method capable of promoting the germination ability of the aged soybean seeds by regulating the antioxidant defense and gluconeogenesis. Germination test revealed that UWT significantly increased the germination rate and seedlings’ establishment of the soybean seeds stored for 12 months, although insignificantly impacting the vigor of fresh (stored for 1 month) and short-term stored (for 6 months) seeds. Further biochemical analysis revealed that UWT decreased the hydrogen peroxide (H_2_O_2_), O_2_⋅^–^, and malondialdehyde contents in the aged soybean seeds during early germination. Consistently, UWT prominently elevated the activities of superoxide dismutase, catalase, and acetaldehyde dehydrogenase, and also the corresponding gene expressions. Besides, the soluble sugar content of UWT was significantly higher than that of the untreated aged seeds. Analysis of enzyme activity showed UWT significantly upregulated the activities of several key enzymes in gluconeogenesis and the transcription levels of corresponding genes. Moreover, UWT enhanced the invertase activity within aged seeds, which was responsible for catalyzing sucrose hydrolysis for forming glucose and fructose. In summary, UWT improved germination and seedlings establishment of aged soybean seeds by regulating antioxidant defense and gluconeogenesis. This study expands the application of ultrasonication in agricultural production and further clarifies the physiological and molecular mechanisms of the aged seed germination, aiming to provide theoretical and practical guidance for seed quality and safety.

## Introduction

Originated in Asia, soybean (*Glycine max* L.) is an important oilseed crop globally (Lam et al., 2010; Schulte et al., 2017). The soybean-planting area is increasing on a yearly basis in China (Yang et al., 2014; Liu et al., 2017). In spite of this, China still remains the world’s major soybean importer. To satisfy the growing demands for vegetable protein, soybean oil, and soybean food, it is imperative to further improve the soybean yield.

Seed germination is the first and paramount stage in the plant growth cycle. The physiological seed germination and seedling emergence are crucial to plant development and crop yield formation (Rajjou et al., 2012; Kan et al., 2016; Rubio de Casas et al., 2017). Generally, it is believed that the seed coat breakthrough by radicle is a sign of germination completion (Shu et al., 2015). After that, the radicle and embryo continue to grow to enter the seedling establishment stage. By following seedlings establishment, the seedlings transit from heterotrophic to autotrophic state (Eastmond et al., 2015; Zhang et al., 2019). The driving energy for both the seed germination and seedling establishment processes is provided by the seed stored materials (Chen and Thelen, 2010).

Triacylglycerol (TAG) is an efficient source of carbon and energy during oil-seed germination (Theodoulou and Eastmond, 2012). During early seed germination, triacylglycerol lipase (TAGL) hydrolyzes the TAG stored in oil to produce fatty acids (FAs) and glycerol. Glycerol kinase (GK) is responsible for catalyzing the glycerol conversion into glycerol 3-phosphate and generates sucrose for seed germination by entering the gluconeogenic pathway (Allen et al., 2015). FAs enter the glyoxylate cycle *via* the ATP-binding cassette (ABC) transporters, which are activated by long-chain acyl-CoA synthase (LACS) (Goepfert and Poirier, 2007). FAs produce acetyl coenzyme A (CoA-SH) *via* the β-oxidation pathway and form oxaloacetate by the tricarboxylate or glyoxylate cycle, which is then converted into sucrose *via* gluconeogenesis (GNG) (Larson et al., 2010; Hurlock et al., 2015). However, the overlong storage time and unfavorable storage environment will greatly inhibit seed germination and seedling establishment, thereby leading to poor-field seedling emergence and substantially affecting soybean quality and yield (Rajjou and Debeaujon, 2008; Nonogaki et al., 2010; Rajjou et al., 2012). Lipid oxidation is an important reason for the deteriorated vigor of oilseeds during storage (Theodoulou and Eastmond, 2012). In addition, the oxidation of unsaturated FAs in oil seeds induces extensive accumulation of reactive oxygen species (ROS), which causes damage to protein, membrane structure, and cell tissues, resulting in the loss of seed vigor (Clemente and Cahoon, 2009). Moreover, the activities of the antioxidant enzyme decreased significantly during seed storage, which further reduced the active oxygen scavenging ability (Lin et al., 2017). Besides, the block of TAG catabolism during early germination is an important reason for the declined vigor of aged soybean and sunflower seeds (Zhou et al., 2019; Huang et al., 2021a). Hence, it is imperative to develop an effective method to improve the seed vigor of aged crop seeds.

Scholars focus on the improvements of germination and seedling establishment from aged crop seeds, and various seed pretreatment methods, including physical, chemical, and biological ways have been well-established. Among them, physical methods are widely known because of the characteristics of environmental protection and efficiency. Seed physical treatments mainly include mechanical shelling, sand burial, electromagnetic process, ultrasonic, and laser treatment (Liu X. et al., 2016). Ultrasound is a sound wave with a frequency above 20 kHz and ultrasonic waves treatment (UWT) is now widely used in seed pretreatment, showing many advantages such as novelty, simple operation, low price, and environmental friendly (Chen et al., 2013; Ramteke, 2015). The role of UWT in improving the crop seed germination has been reported in rice (Li et al., 2010), oilseed rape (Zhao et al., 2012), kidney bean (Qian et al., 2004), common yarrow (Mirshekari et al., 2013), and tall fescue (Liu X. et al., 2016). In addition, UWT has also been demonstrated to improve the resistance to cadmium and salt stresses during crop seed germination (Yang and Guo, 2011). UWT might affect seed germination ability mainly through regulating protein denaturation, endogenous phytohormones balance, and cell damage repair (Li et al., 2010; Ramteke, 2015). Nonetheless, most of these studies focused only on germination rather than the seedling establishment of the aged seeds. Moreover, the underlying mechanisms of UWT-induced improvement of seed vigor in aged crop seeds need further investigation.

To the present, the research and application of UWT have not received sufficient attention. UWT in crop production has huge potential regarding feasibility and diversity. In this study, we found that the UWT could eliminate the ROS accumulation by activating the antioxidant system, promoting GNG, and thereby improving the germination and seedlings’ emergence of naturally aged soybean seeds. We hope that this study can expand the application of ultrasonication in agricultural production and further clarify the physiological and molecular mechanisms of aged seed germination, aiming to provide theoretical and practical guidance for seed quality and safety.

## Materials and Methods

### Materials

The soybean (*Glycine max* L.) seeds of “Zhexian NO.9,” produced in the experimental farm of Zhejiang Academy of Agricultural Science, were used in our study. The seeds were stored in the airtight dry container at room temperature for 1, 6, 12, 18, and 24 months for naturally aging treatment (NAT), respectively.

### Accelerated Aging Test

The 1-month stored soybean seeds were used for the accelerated aging test (AAT) according to the method of Missaoui and Hill (2015) with small modifications. Briefly, 100 soybean seeds samples were placed into a seed-aging box and incubated at 45°C for 48 h under 100% relative humidity. After that, processed seeds were subjected to 2 days of drying under room temperature prior to the germination test.

### Ultrasonic Waves Treatment

Preliminary tests were conducted to determine the condition for ultrasonic treatment (Supplementary Table 1). Then, soybean seeds were subject to ultrasonic treatment for 15 min with the instrument ultra sonicator (BOKE, BKE-1002DHT; dimension, L × W × D: 300 mm × 240 mm × 150 mm; power, 100 W; and frequency, 40 kHz). Distilled water was used as the control.

### Seed Germination and Seedling Emergence Tests

Soybean seeds were disinfected with sodium hypochlorite solution (0.1%) for 15 min before the seed germination test. Briefly, rolled towels placed with 50 soybean seeds for each treatment were soaked with purified water and incubated at 25°C. The number of germinated seeds was counted at 0–7 days of germination. At 0, 1, and 3 days of germination, the soybean seeds were collected and used for further biochemical analysis. Soybean seeds were planted in sand at 25°C under the 12/12 h light/dark cycle. On day 7, the seedling establishment ratio (SER) was measured based on the formula SER = Number of normal seedlings/Number of tested seeds (Zhang et al., 2007). The seed germination and seedling emergence tests were performed with four biological replications.

### Hydrogen Peroxide and Superoxide Anion Analysis

Determination of hydrogen peroxide (H_2_O_2_) and superoxide anion (O_2_⋅^–^) content was analyzed using spectrophotometry. Briefly, 0.3 g of seed samples were homogenized in 3 ml of acetone and centrifuged at 12,000 rpm for 15 min. Later, 1 ml of supernatant was collected and mixed with 0.1 ml of titanium sulfate solution (5%) and 0.2 ml of ammonia water (25%). After centrifugation again at 12,000 rpm for 15 min, the precipitate was collected and dissolved with 3 ml of sulfuric acid (2 mM). Seeds sample was incubating in 3 ml of a reaction mixture [10 mM sodium phosphate buffer (pH 7.8), 10 mM sodium azide (NaN_3_), and 0.05% (m/v) nitroblue-tetrazolium (NBT)]. Finally, H_2_O_2_ level was measured based on supernatant absorbance value (OD) at 390 nm with the method of Hasanuzzaman et al. (2021), and O_2_⋅^–^ level was calculated based on the absorbance of the supernatant at 580 nm according to the method of Soares et al. (2021). All the samples were assayed with four biological replicates.

### Lipid Peroxidation Analysis

Malondialdehyde (MDA) content was measured for the determination of lipid peroxidation with the method of Padilla et al. (2021). Briefly, the seeds sample was homogenized with 0.05 M phosphate buffer (pH 7.8) and centrifuged at 10,000 rpm for 15 min. The supernatant was mixed with 1 ml of 5% (w/v) thiobarbituric acid-trichloroacetic acid solution and used for spectrophotometric determination. MDA level (μmol g^–1^ FW) were calculated by the formula MDA = [(OD_532_−OD_600_) × A × C]/(0.155 × W). In the formula, A and C corresponded to the volume of the reaction solution and extraction solution, respectively, and W represented the fresh weight of the sample. The analysis of MDA was performed with four biological replicates.

### Antioxidant Enzymes

Antioxidant enzymes analysis was performed based on the method of Soares et al. (2021). Potassium phosphate buffer (pH 7.5) was utilized for seeds homogenization. The homogenates were centrifuged at 12,000 × *g* for 20 min to collect supernatant for the measurement of activities of superoxide dismutase (SOD), peroxidase (POD), catalase (CAT), ascorbate peroxidase (APX), and acetaldehyde dehydrogenase (ALDH). The analysis of antioxidant enzymes was performed with four biological replicates.

### Sugars Quantification

Anthrone-H_2_SO_4_ colorimetry was performed to determine soluble sugar content in soybean seeds according to the method of Yi and Dong (2008) with four biological replicates. Sucrose level was measured by the resorcinol method and estimated by absorbance value at 480 nm (Shi et al., 2016). In addition, fructose content was measured by the method of Cai et al. (2016). The glucose level in soybean seed was measured through high-performance liquid chromatography (HPLC) according to the method of Bailly et al. (2010).

### Adenosine Triphosphate and Energy Charge Analysis

Seed samples (2 g) were homogenized into powder and extracted with 0.5 mol/l perchloric acid (10 ml) at 4°C for 5 min. The homogenization was centrifuged at 12,000 rpm for 10 min, and the supernatant (5 ml) was collected and filtered through a 0.22 μm membrane filter. The determinations of ATP and energy charge were performed by HPLC with the method of Emami and Kempken (2019). Data were expressed as the average values from four biological replicates.

### Assay of Triacylglycerol and Glycometabolism Metabolism-Involved Enzymes Activity

Enzyme-linked immunosorbent assay (ELISA) was used to determine the activities of lipase (LIPG), GK, α-glycerol phosphate oxidoreductase (GPDH), citrate synthase (CS), aconitase (ACO), isocitrate lyase (ICL), malate synthase (MS), malate dehydrogenase (MDH), succinate dehydrogenase (SDH), fumarase (FUM), malate degydrogenase (MDH), phosphoenolpyruvate carboxykinase (PCK), enolase, phosphoglycerate kinase (PGK), glyceraldehyde-3-phosphate dehydrogenase (GAPDH), aldolase, fructose-1,6-bisphosphatase (FBPase), UDPG1c pyrophosphorylase (UDPG PPase), sucrose synthase (SUSY), sucrose-6-P synthase (SPS), sucrose phosphatase (SPP), pyrophosphatase (PPase), and invertase (INV) with the ELISA kit (Mlbio, Shanghai, China). Briefly, soybean seeds were extracted using the extraction buffer, and then the chromogenic reaction was carried out with the Chromogen Solution. The color change was tested by absorbance value at 450 nm. The activities of TAG and glycometabolism-related enzymes in soybean seeds were then determined by comparing the OD of these samples to the standard curve.

### Real Time Quantitative Polymerase Chain Reaction

The PrimeScript RT Reagent Kit (Vazyme, Nanjing, China) was employed to extract RNA from seeds and prepare cDNA through reverse transcription. The CFX96 Touch Real-Time PCR system (Biorad) was utilized for RT-qPCR procedures by using the ChamQ SYBR qPCR Master Mix (Vazyme, Nanjing, China). Supplementary Table 2 presents all the primers utilized in this study, with *GmTubulin* being the internal control. The fold change of expression (FC) was determined with the formula of FC = EΔCt, where E stands for the average gene amplification efficiency, ΔCt indicates the difference in average Ct values for all biological replicates between two compared samples. The normalized results were displayed in a form of mean SD. The RT-PCR assays were performed with four independent biological replicates and three technical replicates.

### Statistical Analysis

Data were statistically analyzed by one-way ANOVA using Statistical Analysis System (SAS) software. Besides, the least significant difference at *p* < 0.05 (LSD_0_._05_) was adopted for multiple comparisons. Percentage data were converted to arcsintrans values by y = arcsin [sqrt (x/100)] before statistical analysis.

## Results

### Storage Significantly Inhibited Soybean Seed Germination and Seedling Emergence

**FIGURE 1:**
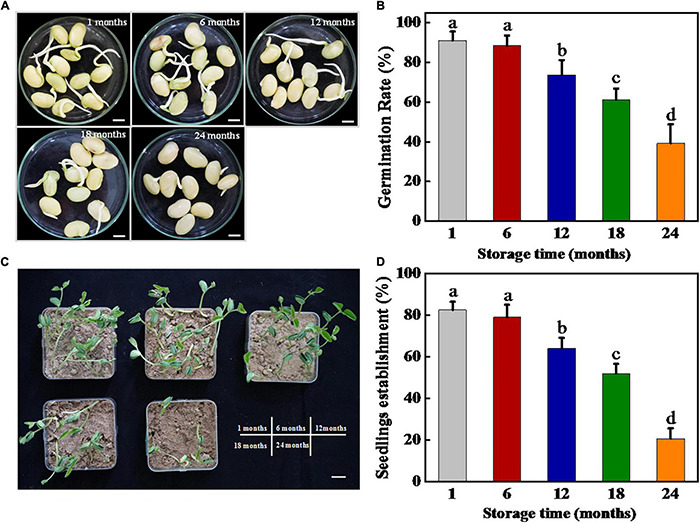
Storage significantly inhibited soybean seed germination and seedling establishment. **(A)** Images of soybean seeds at 3 days of germination. Scale bar, 10 mm. **(B)** Germination rates of soybean seeds at 5 days of germination. **(C)** Images of seedling emergence from soybean seeds at 5 days postsowing. Scale bar, 600 mm. **(D)** Seedling emergence rates of soybean seeds at 5 days postsowing. Different lowercase letters indicate significant differences between different treatments (*p* < 0.05).

The harvested soybean seeds were stored for 1, 6, 12, 18, and 24 months, respectively, and then subjected to germination test and seedling establishment (Figure 1). The results showed that the soybean seeds maintained a high-germination rate of 90% following both 1 and 6 months of storage. Long-term storage (12, 18, and 24 months) significantly reduced the germination rate of soybean seeds. On day 7 of germination, 12 and 18 months stored sunflower seeds achieved 71 and 65.5% germination rates, respectively, while 66% of soybean seeds lost their germination capacity following 24 months of storage.

Similarly, the soybean seedling establishment rate showed a substantial decrease with the increase in storage time. No significant differences in seedling establishment rate were observed between soybean seeds stored for 1 and 6 months, reaching approximately 80%. By contrast, for the seeds stored for 12, 18, and 24 months, the seedling establishment rates decreased significantly, which were 61.50, 49.75, and 20.50%, respectively. Besides, the seedling establishment rates were all lower than the germination rates after storing for different times. In summary, the aforementioned results revealed that natural aging significantly decreased the germination and seedling establishment ability of soybean seeds.

### Ultrasonic Waves Treatment-Enhanced Naturally Aged Soybean Seed Germination and Seedling Establishment

**FIGURE 2:**
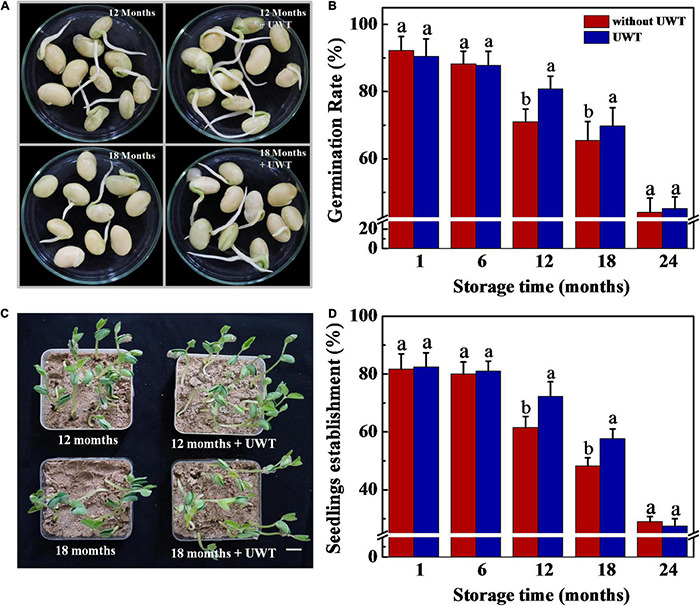
Ultrasonic waves treatment promoted germination and seedling establishment of NAT soybean seeds. **(A)** Images of soybean seeds at 3 days of germination. Scale bar, 10 mm. **(B)** Germination rates of soybean seeds at 5 days of germination. **(C)** Images of seedling emergence from soybean seeds at 5 days post-sowing. Scale bar, 600 mm. **(D)** Seedling emergence rates of soybean seeds at 5 days postsowing. Soybean seeds stored for 1, 6, 12, 18, and 24 months were used for analysis. NAT, naturally aging treatment; UWT, ultrasonic waves treatment. Different lowercase letters indicate significant differences between different treatments (*p* < 0.05).

The result proved that UWT exhibited a positive effect on germination and seedling establishment from NAT soybean seeds (Figure 2). Following 12 and 18 months of storage, the UWT seeds exhibited significantly higher rates of germination and seedling emergence in comparison with the control. However, no remarkable difference in seed germination and seedling emergence was detected between NAT and NAT + UWT treatments in soybean seeds stored for 1, 6, and 24 months.

Given the role of UWT in promoting seed vigor of soybean seeds following the storage process, we further explored the effect of UWT on the germination of artificially aged soybean seeds (Supplementary Figure 1). As a result, artificial accelerated aging remarkably decreased soybean seed germination and seedling emergence rates. Nevertheless, UWT made no significant effect on artificially aged soybean seed germination or seedling emergence. Generally, we speculated that the germination of NAT and AAT soybean seeds might respond differently to UWT. After proving the role of UWT in improving the seed vigor of NAT soybean seeds, only 12 months of stored seeds were adopted for further tests.

### Ultrasonic Waves Treatment Decreased the Contents of Hydrogen Peroxide, Superoxide Anion, and Malondialdehyde in Aged Soybean Seeds During Early Germination

**FIGURE 3:**
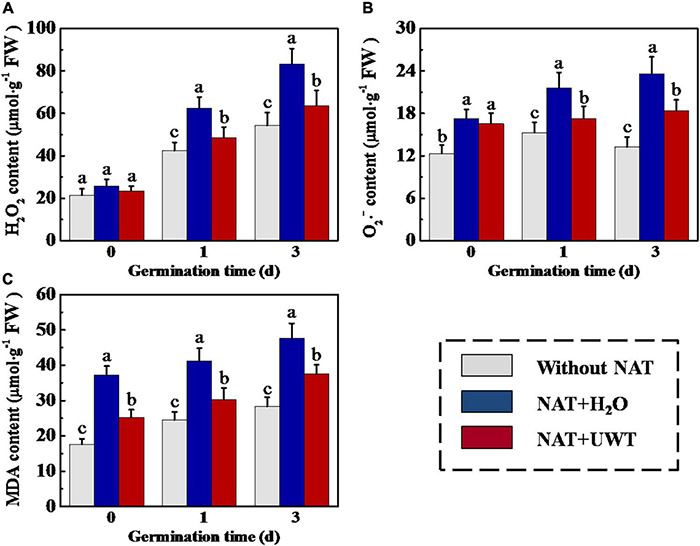
Ultrasonic waves treatment decreased the levels of H_2_O_2_ **(A)**, O_2_⋅^–^ **(B)**, and MDA **(C)** of soybean seeds during early germination. Without NAT, fresh seeds without naturally aging treatment (NAT); NAT + H_2_O, Seeds stored for 12 months with H_2_O; NAT + UWT, NAT seeds with ultrasonic waves treatment (UWT). H_2_O_2_, hydrogen peroxide; O_2_⋅^–^, superoxide anion; MDA, malondialdehyde; Four biological replicates for each treatment were set in quantification of H_2_O_2_, O_2_⋅^–^, and MDA. Different lowercase letters indicate significant differences between different treatments (*p* < 0.05).

Overaccumulation of ROS and MDA in soybean seeds during storage was an important reason for the deterioration of seed vigor. To study the mechanism whereby UWT improved the vigor of aged soybean seeds, we determined the H_2_O_2_, O_2_⋅^–^, and MDA contents in soybean seeds during early germination. As shown in Figure 3, UWT effectively alleviated the overaccumulation of ROS and MDA in aged soybean seeds. On 1 and 3 days of germination, the H_2_O_2_, O_2_⋅^–^, and MDA contents of NAT + H_2_O were significantly higher than those without NAT seeds. While NAT + UWT seeds exhibited significantly lower contents of H_2_O_2_, O_2_⋅^–^, and MDA than the NAT group on 1 and 3 days of germination. Besides, UWT also significantly reduced the MDA content of aged soybean seeds on 0 day of germinated (Figure 3).

### Ultrasonic Waves Treatment Upregulated the Activities of Antioxidases and Abundance of Corresponding-Genes Expressions in Aged Soybean Seeds During Early Germination

**FIGURE 4:**
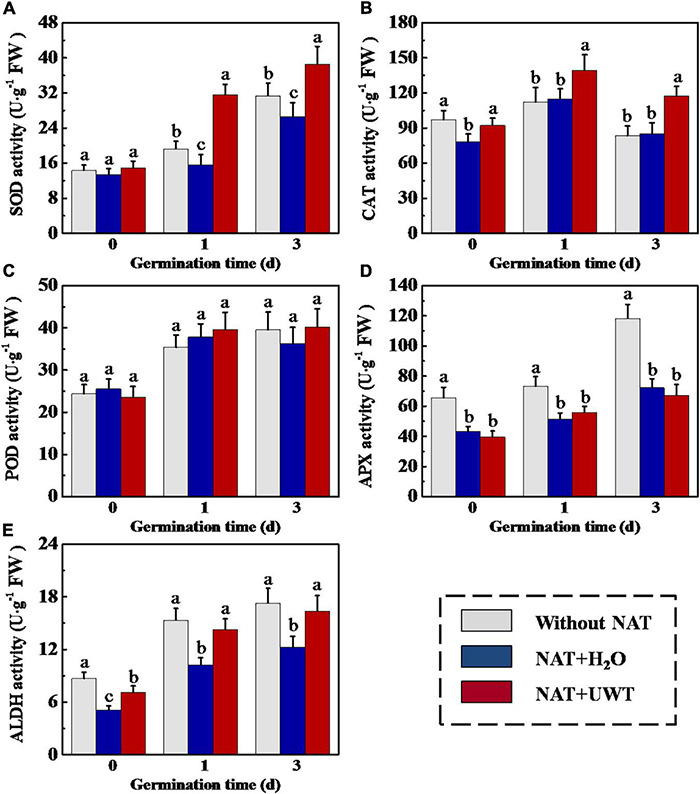
Ultrasonic waves treatment increased the activities of SOD **(A)**, CAT **(B)**, POD **(C)**, APX **(D)**, and ALDH **(E)** of aged soybean seeds during early germination. Without NAT, fresh seeds without naturally aging treatment (NAT); NAT + H_2_O, Seeds stored for 12 months with H_2_O; NAT + UWT, NAT seeds with ultrasonic waves treatment (UWT). SOD, superoxide dismutase; CAT, catalase; POD, peroxidase; APX, ascorbate peroxidase; ALDH, acetaldehyde dehydrogenase. Different lowercase letters indicate significant differences between different treatments (*p* < 0.05).

**FIGURE 5:**
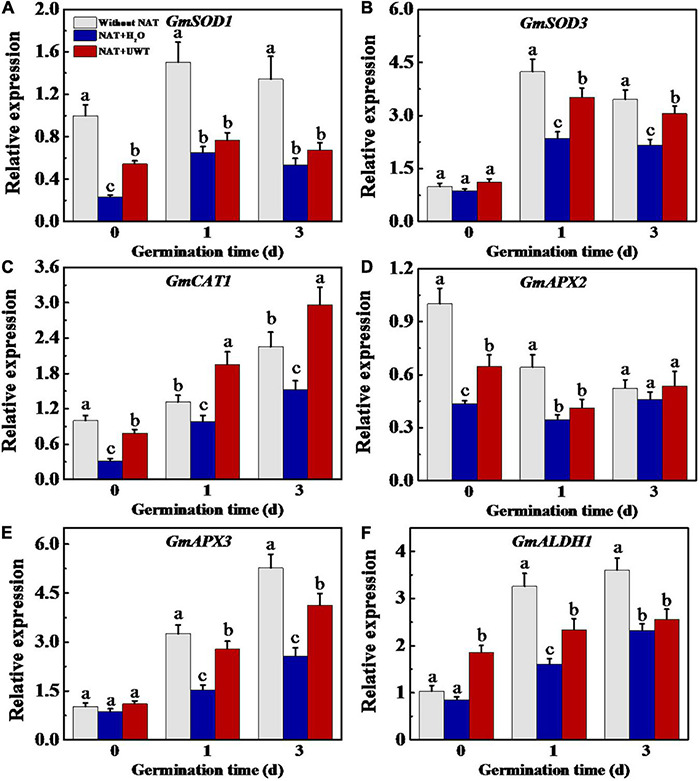
Ultrasonic waves treatment increased the transcriptional levels of *GmSOD1* **(A)**, *GmSOD3* **(B)**, *GmCAT1* **(C)**, *GmAPX2* **(D)**, *GmAPX3* **(E)**, and *GmALDH1* **(F)** of aged soybean seeds during early germination. Without NAT, fresh seeds without naturally aging treatment (NAT); NAT + H_2_O, Seeds stored for 12 months with H_2_O; NAT + UWT, NAT seeds with UWT. The RT-PCR assays were performed with four independent biological replicates and three technical replicates. Different lowercase letters in the same column indicate significant differences between different treatments (*p* < 0.05).

Considering the effect of UWT on the ROS and MDA levels in aged soybean seeds during early germination, we further evaluated the activities of antioxidant enzymes and ALDH. The results revealed that NAT dramatically lowered the activities of SOD, APX and ALDH as compared with fresh seeds during early germination (Figures 4A,D,E). UWT significantly enhanced the SOD, CAT and ALDH activities in NAT soybean seeds on 1 and 3 d of germination (Figures 4A,B,E). However, no significant difference in POD activity was observed between NAT + H_2_O and NAT + UWT during early germination (Figure 4C). Consistently, the qPCR analysis also revealed significantly lower expressions of corresponding genes in NAT soybean seeds in comparison with fresh seeds, including *GmSOD1*, *GmSOD3*, *GmCAT1*, *GmAPX2*, *Gm APX3*, and *GmALDH1* (Figure 5). Besides, significantly higher transcriptional levels of *GmSOD1, GmAPX2*, and *GmALDH1* have been observed in NAT + UWT seeds compared with NAT + H_2_O treatment at 0 day of germination. UWT also increased the transcriptions of *GmSOD3*, *GmAPX3*, and *GmCAT1* in NAT soybean seeds at 1 and 3 days of germination (Figure 5).

### Ultrasonic Waves Treatment Increased the Soluble Sugars and Adenosine Triphosphate Contents of Aged Soybean Seeds During Early Germination

**FIGURE 6:**
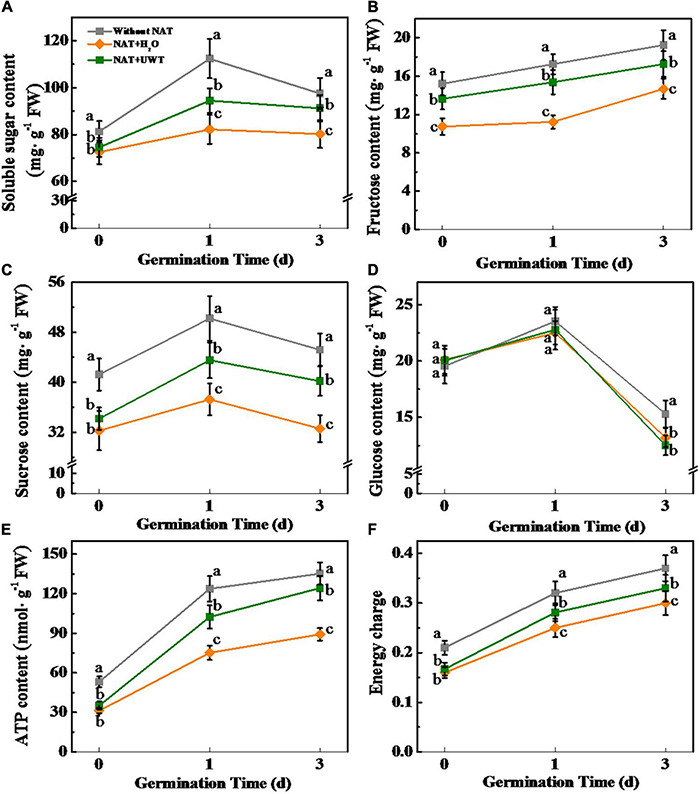
Ultrasonic waves treatment increased the contents of soluble sugar **(A)**, fructose **(B)**, sucrose **(C)**, Glucose **(D)**, ATP **(E)**, and energy charge **(F)** of aged soybean seeds during early germination. Without NAT, fresh seeds without naturally aging treatment (NAT); NAT + H_2_O, Seeds stored for 12 months with H_2_O; NAT + UWT, NAT seeds with UWT. Different lowercase letters in the same column indicate significant differences between different treatments (*p* < 0.05).

During seed imbibitions, the soluble sugars are the primary energy source for early germination and seedlings establishment, including sucrose, glucose, and fructose (Eastmond et al., 2015). To better investigate the effect of UWT in improving the germination and seedling establishment of aged soybean seeds, we further determined the levels of soluble sugar, sucrose, glucose, and fructose (Figure 6). Sugars quantification revealed that NAT markedly lowers the contents of soluble sugar, sucrose, and fructose during early germination time. Interestingly, UWT significantly elevated the levels of soluble sugar, sucrose, and fructose in NAT soybean seeds. Moreover, significantly higher levels of ATP and energy charge were detected in NAT + UWT seeds as compared with NAT + H_2_O seeds (Figures 6E,F). Nevertheless, the glucose content was not significantly affected by NAT or UWT during soybean seed germination (Figure 6D).

### Ultrasonic Waves Treatment Induced the Activities of Gluconeogenesis-Involved Enzymes in Aged Soybean Seeds During Early Germination

**FIGURE 7:**
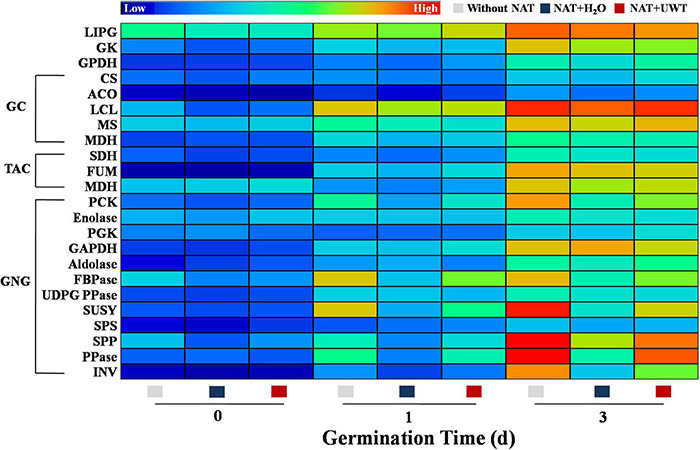
Ultrasonic waves treatment made an effect on the activities of enzymes related to triacylglycerol metabolism of aged soybean seeds during early germination. Without NAT, fresh seeds without naturally aging treatment (NAT); NAT + H_2_O, Seeds stored for 12 months with H_2_O; NAT + UWT, NAT seeds with ultrasonic waves treatment (UWT). LIPG, Lipase; GK, Glycerol kinase; GPDH, α-glycerol phosphate oxidoreductase; CS, citrate synthase; ACO, aconitase; ICL, isocitrate lyase; MS, malate synthase; MDH, malate dehydrogenase; SHD, succinate dehydrogenase; FUM, fumarase; PCK, phosphoenolpyruvate carboxykinase; PGK, phosphoglycerate kinase; GAPDH, glyceraldehyde-3-phosphate dehydrogenase; FBPase, fructose-1,6-bisphosphatase; UDPG PPase, UDPG1c pyrophosphorylase; SUSY, sucrose synthase; SPS, sucrose-6-P synthase; SPP, sucrose phosphatase; PPase, pyrophosphatase; INV, invertase. The heat map was created by the Illustrator software, and the enzyme activities levels from lowest to highest were represented by different colors from blue to red in the entire database.

During early oil-seed germination, TAGs were decomposed into soluble sugars through complex metabolism, thus providing energy for seed germination. The catabolism of TAGs involved several key enzymes, mainly including LIPG, GK, GPDH, CS, ACO, LCL, MS, MDH, PCK, Enolase, PGK, GAPDH, Aldolase, FBPase, UDPGPPase, SUSY, SPS, SPP, PPase, and INV. This study revealed that the activities of TAG catabolism-related enzymes continued to elevate during early germination time (Figure 7). In addition, NAT significantly inhibited the activities of a couple of key GNG-involved enzymes. Obviously, NAT failed to affect the activities of LIPG, GK, GPDH, and enzymes involved in GC and TAC. However, what is noteworthy is that UWT drastically enhanced the activities of several key GNG-related enzymes in the aged soybean seeds germinated for 1 and 3 days, including PCK, FBPase, SUSY, SPP, PPase, and INV. Similarly, no significant differences in the activities of LIPG, GK, GPDH, and enzymes involved in GC and TAC were detected between the NAT + UWT and the NAT + H_2_O treatment (Figure 7).

### Ultrasonic Waves Treatment Upregulated the Transcription of Gluconeogenesis-Involved Genes in Aged Soybean Seeds During Early Germination

**FIGURE 8:**
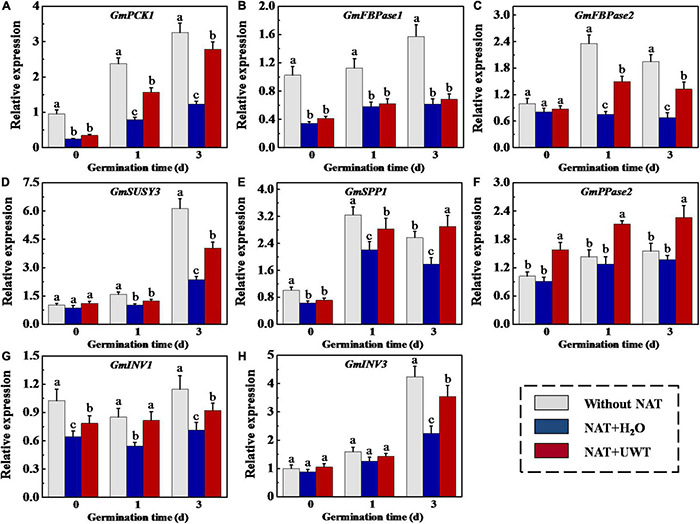
Ultrasonic waves treatment increased the transcriptional levels of *GmPCK1* **(A)**, *GmFBPase1* **(B)**, *GmFBPase2* **(C)**, *GmSUSY3* **(D)**, *GmSPP1* **(E)**, *GmPPase2* **(F)**, *GmINV1* **(G)**, and *Gm INV3* **(H)** of aged soybean seeds during early germination. Without NAT, fresh seeds without naturally aging treatment (NAT); NAT + H_2_O, Seeds stored for 12 months with H_2_O; NAT + UWT, NAT seeds with ultrasonic waves treatment (UWT). The RT-PCR assays were performed with four independent biological replicates and three technical replicates. Different lowercase letters in the same column indicate significant differences between different treatments (*p* < 0.05).

The gene expression analysis showed that the transcriptions of *GmPCK1*, *GmFBPase1*, *GmFBPase2*, *GmSUSY3*, *GmSPP1*, *GmINV1*, and *GmINV3* were downregulated by NAT. While NAT made no effect on *GmPPase2* expression during soybean seed germination. It should be noted that the regulation of UWT on GNG-related gene expression was opposite to that of NAT. UWT remarkably increased the transcriptions of *GmPCK1*, *GmFBPase2*, *GmSPP1*, and *GmINV1* at 1 and 3 days of germination. Besides, the transcriptional levels of *GmSUSY3* and *GmINV3* in NAT + UWT seeds were significantly high than those in NAT seeds (Figure 8).

## Discussion

Seeds are the basis of agricultural production, and high seed vigor plays an important role in crop growth and yield (Nonogaki et al., 2010; Ellis, 2011). The loss of seed vigor is usually accompanied by seed storage, which is tightly related to seed property, storage period, and conditions. Consequently, exploring ways to enhance the ability of germination and seedling emergence following seed storage provides profound economic and ecological significance. In the present study, the natural storage treatment remarkably inhibited the germination and seedling emergence of soybean seeds. The excessive ROS accumulation and block of GNG might be important reasons for the deteriorated vigor of NAT soybean seeds. UWT prominently increased the rates of germination and seedling establishment from NAT soybean seeds, which might be in close association with the antioxidant defense and GNG.

### Ultrasonic Waves Treatment Improved the Germination and Seedling Emergence in Naturally Aging Treatment Soybean Seeds

In agricultural production, improper storage methods or prolonged storage time often led to seed deterioration, which, in turn, inhibited the field seedling emergence (Yin et al., 2014, 2015; Fleming et al., 2017). Accordingly, a study on seed pretreatment methods to enhance the vigor of aging seeds from the perspectives of molecular mechanism and production application is a worthy objective of concern in agricultural production. Maffei (2014) reported that the magnetic field treatment could activate the absorption of water and nutrients, influence the cell membrane composition and integrity, thereby promoting seed germination. Similarly, Hu and Zhou (2014) found that novel-hydrated graphene ribbon accelerated the germination speed and improved the seedling characteristic from stored wheat seeds by enhancing the integrity of cell membranes and increasing the contents of soluble sugars, amino acids, and FAs. Cold plasma application could facilitate rapeseed germination with drought stress (Li et al., 2015). Besides, a nanopriming technology was developed by Mahakham et al. (2017) for promoting seed vigor from stored rice seeds with photosynthesized silver nanoparticles. This study found that ultrasonic treatment could boost the germination and seedling emergence of NAT soybean seeds, but failed to enhance the vigor of artificially aged soybean seeds.

Ultrasound, as a sound wave with a frequency above 20 kHz, is characterized by good directivity and penetration (Suslick, 1990). UWT of seeds is a novel physical method featuring simple operation, low cost, and eco-friendliness. Several reports have revealed the function of ultrasonic treatment on seed vigor improvement (Chen et al., 2012; Ran et al., 2015). UWT could activate the transportation of water and oxygen, the release of hydrolytic enzymes, accelerate the decomposition of protein and starch in the endosperm, and lead to improved germination of cereal crop seeds (Patero and Augusto, 2015). Nevertheless, UWT may also lead to nutrient leakage to adversely influence seed germination (Wang et al., 2012). Besides, Liu J. et al. (2016) proved the role of UWT on germination improvement in tall fescue and *Russian wildrye* aged seeds, but the underlying mechanism remained unclear. However, most of these studies focused on germination rather than seedling emergence of the aged seeds, especially in aging oil seeds. Further study is required to explore the intrinsic mechanism of UWT-induced promotion on seed germination ability. This study extended the practical uses of UWT, suggesting that UWT could reverse the inhibition of seed vigor of soybean induced by natural aging.

### Ultrasonic Waves Treatment Enhances the Antioxidant System of Naturally Aged Soybean Seeds

Reactive oxygen species exert a vital role in cellular metabolism, especially affecting the dormancy and aging of seeds (Oracz and Karpinski, 2016; Sachdev et al., 2021). During the seeds storage, the decrease of activities of antioxidant enzymes, lipid peroxidation, and substantial accumulation of ROS, MDA resulted in the deterioration of seed vigor (Corbineau and Bailly, 2007). Our previous study reported that naturally aging significantly increased the accumulations of H_2_O_2_, O_2_⋅^–^, and MDA and resulting in the acceleration of seed deterioration (Huang et al., 2021b). ROS has a dual function, which plays a positive role in seed development, germination, and resistance to environmental stress at appropriate concentrations, while generating toxic effects on the cells and plant tissues at excessive concentrations (Barba-Espin et al., 2011; Oracz et al., 2015). Herein, compared with the higher contents of ROS (H_2_O_2_, O_2_⋅^–^) and MDA in NAT soybean seeds than the fresh seeds, NAT + UWT treatment effectively alleviated the overproduction of ROS and MDA during 1 and 3 days germination. It is crucial to maintain ROS homeostasis during seed germination, so as to guarantee seed germination as well as subsequent seedling emergence (Yin et al., 2015; Deng et al., 2017). This study proposed that UWT could enable better ROS scavenging ability in seeds during early germination than the control. This is similar to the previous study showing that maintaining ROS homeostasis is one of the mechanisms underlying UWT improved seed germination under abiotic stress (Chen et al., 2013).

Antioxidases, mainly including POD, SOD, APX, and CAT, are crucial to the ROS scavenging system (Mittler, 2006; Hasanuzzaman et al., 2020; Sachdev et al., 2021). Lin et al. (2017) found that the activities of POD, CAT, APX, and SOD decreased gradually during paddy seed storage, showing positive correlations with the seed vigor. Similarly, our results revealed significantly lower activities of SOD, ALDH, and corresponding genes expressions in NAT soybean seeds compared with fresh seeds. By contrast, the antioxidant enzymes activities are closely related to ROS level in soybean seeds; to be specific, NAT + UWT seeds showed significantly increased SOD, ALDH, and CAT activities relative to NAT + H_2_O seeds. Consistently, the gene expression analysis revealed that UWT significantly increased the transcription levels of *GmSOD1*, *GmSOD3*, *GmCAT1*, *GmAPX2*, and *GmALDH1* in NAT soybean seeds at 1 and 3 days of germination. The above results were consistent with the previous study reporting that ultrasonic treatment could improve the germination of aged Russian wild rye (*Psathyrostaehys juncea* Nevski) and tall fescue (*Festuca arundinacea*) seeds by regulating antioxidant defense (Liu J. et al., 2016), showing that UWT had a certain effect on the efficacy of antioxidant enzyme system in aged seeds during imbibitions. The successful activating of antioxidant defense by UWT was also reported by several other studies (Chen et al., 2012; Yang et al., 2015).

### Ultrasonic Waves Treatment Promoted the Gluconeogenesis in Aged Soybean Seeds

Admittedly, soluble sugars, mainly sucrose and fructose, are the primary energy source during early germination (Quettier et al., 2008; Eastmond et al., 2015). Soybean seeds are abundant in TAG, and TAG catabolism provides substantial energy for the germination and seedling establishment processes (Theodoulou and Eastmond, 2012). The mobilization and conversion of stored TAG into soluble sugars involve multiple pathways and subcellular fractions (Li-Beisson et al., 2010). Triacylglycerol lipase (LIPG) hydrolyzes TAGs stored in oil to produce FAs and glycerol (Basnet et al., 2016). GK catalyzes the conversion of glycerol into glycerol 3-phosphate and enters the gluconeogenic pathway. FAs produce CoA-SH *via* the β-oxidation process, generating sucrose *via* the glyoxylate cycle, and GNG (Eastmond, 2006). In this study, NAT significantly inhibited soybean seed germination and seedling emergence, while UWT effectively alleviated the NAT-induced inhibition of soybean seed vigor. Further analyses demonstrated that NAT significantly lowered the activities of several key gluconeogenic enzymes such as PCK, FBPase, SUSY, and SPP, and also the corresponding gene expression levels. Consequently, the soluble sugar content during early germination declined drastically. PCK catalyzes the production of phosphoenolpyruvate from oxaloacetate, which represents the rate-limiting step in the metabolic pathway (Penfield et al., 2012). Fructose-1,6-bisphosphatase (FBPase) functions to catalyze the conversion of fructose-1,6-bisphosphate to fructose-6-phosphate (Graham, 2008; Quettier et al., 2008). SUSY and SPP are in charge of catalyzing UDPGlc conversion into sucrose, which then subsequently produces fructose and glucose by invertase (Kaur and Hu, 2009; Ma et al., 2011). Pyrophosphatase (PPase) is another key enzyme involved in GNG, which catalyzed the generation of phosphate from cytosolic pyrophosphate. A PPase-deficient *Arabidopsis* mutant (*fugu5*) shows a poor seedling emergence rate due to the block of sucrose production, even though the total glyoxysome-associated processes except for b-oxidation can be completed (Ferjani et al., 2011). In this study, UWT significantly increased the activity of PPase and *GmPPase2* expression in NAT soybean seeds, suggesting that NAT treatment suppressed TAG conversion to sucrose, as supported by Zhou et al. (2019) indicating that the blocked FA catabolism accounted for the primary cause of the low-aged soybean seed vigor (Zhou et al., 2019). In contrast, UWT significantly augments the enzymes activities and corresponding-gene expressions associated with GNG, and also the accumulation of soluble sugars, sucrose, and fructose. Interestingly, NAT and UWT did not significantly affect the activities of LIGP, as well as of enzymes related to glyoxylate or tricarboxylate cycle. It was proposed that the inactivation of GNG might be a major reason for the deteriorated vigor of soybean seeds during storage. In addition, UWT could improve soybean seeds germination by weakening the GNG inhibition induced by NAT.

## Conclusion

Overall, our results revealed that UWT had the function of improving the seed germination and seedling emergence from NAT soybean seeds. Naturally aging made a negative effect on glycometabolism and resulted in the block of energy supply for soybean seed germination and seedling emergence. Besides, naturally aging induced the overaccumulation of ROS and MDA and led to damage to seed germination. By applying different approaches, the main mechanisms behind UWT-induced improvement on aged soybean seeds germination were unraveled, namely, upregulation of GNG and the efficiency of the enzymatic antioxidant defense being the main factors behind the higher vigor of UWT-treated soybean seeds. This work sheds more light on the theoretical and practical foundation for the application of UWT in enhancing the safety production of aged crop seeds.

## Data Availability Statement

The original contributions presented in the study are included in the article/Supplementary Material, further inquiries can be directed to the corresponding author/s.

## Author Contributions

YH and DC: conceptualization and resources. YH and GM: investigation. YH and YW: writing—original draft preparation. DC: writing—review and editing. XF and XR: supervision. All authors read and agreed to the published version of the manuscript.

## Conflict of Interest

XR was employed by Zhejiang Nongke Seed Co. Ltd. The remaining authors declare that the research was conducted in the absence of any commercial or financial relationships that could be construed as a potential conflict of interest.

## Publisher’s Note

All claims expressed in this article are solely those of the authors and do not necessarily represent those of their affiliated organizations, or those of the publisher, the editors and the reviewers. Any product that may be evaluated in this article, or claim that may be made by its manufacturer, is not guaranteed or endorsed by the publisher.
