# Supplementary material for: Ultrasonic Waves Regulate Antioxidant Defense and Gluconeogenesis to Improve Germination From Naturally Aged Soybean Seeds
Source: Front Plant Sci. 2022 Mar 28;13:833858. doi: 10.3389/fpls.2022.833858 (PMC8996252; doi:10.3389/fpls.2022.833858)
Supplement: Supplementary file 1 [file Data_Sheet_1.PDF]

Ultrasonic waves regulates antioxidant defense and gluconeogenesis to improve germination from naturally aged soybean seeds

Table S1. The effect of ultrasonic waves treatment (UWT) on soybean seeds germination and seeding establishment.

**Treatment**	**Frequency (kHz)**	**Priming time (min)**	**Germination rate (%)**
Control-1	0	10	70.25±4.257 d*
Control-2	0	15	71.75±2.331 c
Control-3	0	20	68.50±4.256 e
UWT-1	20	10	69.25±3.451 de
UWT-2	20	15	74.75±3.124 b
UWT-3	20	20	64.00±5.124 g
UWT-4	40	10	75.50±3.127 b
UWT-5	40	15	81.75±4.264 a
UWT-6	40	20	72.00±3.594 c
UWT-7	60	10	64.50±4.254 g
UWT-8	60	15	65.75±3.295 f
UWT-9	60	20	60.00±4.551 h

*Values followed by a different letter within a column are significantly different at the 0.05 probability level. Seeds stored for 12 month were used for test. Values are means of four replicates.

Table S2. Primers used in qRT-PCR analysis of genes expression

**Gene name**	**Primer sequence**	
	**Forward primer**	**Reverse primer**
GmTubulin	AACCTCCTCCTCATCGTACT	GACAGCATCAGCCATGTTCA
GmSOD1	TTGCTGGTCTTAAGCCTGGT	GGCAACCATTGGTAGTGTCC
GmSOD3	TCCAACCACCGTAACTGGAT	CAAGGCATGGACATGGAAA
GmCAT1	CCCAGGACCTTTACGATTCA	GGATGTAGAGTTTCCACTCAGGA
GmAPX2	AAGGGTTCTGACCATTTGA	AAGGTAGCTGAAGGAGGC

GmAPX3	AAAAGTTTCATACGCAGAC	ATACCCATACGAGAA
GmALDH1	CATTCCACTTACCGACAC	TCTCCCTCACTAACTCTTG
GmPCK1	ACCTAAGTTACATGGTGCAGTG	CGTGCTCTGTTCCGTTCCTT
GmFBPase1	GTTCTGGAGTTAGGTGCATGG	TTCTCTTCTTTGTCCCAGTGGT
GmFBPase2	GGGGGTTCTGGAGTTAGGTG	TCTTCTTTGTCCCAGTGGTTG
GmSUSY3	TTGATCCCAAGTTCAACATAGC	TTGACTTTGTCTGTGTAAGGGAAA
GmSPP1	CAATGCCCAAGAGGAACTGT	GCATGAAGAATCTTGGGGTTA
GmPPase2	CAACCTTGCCATGACTTCAA	CACATGTTTGGAGGGCATAA
GmINV1	TGAAGGGGAAATTGTCAAGAA	CTTGCCCTGGACTGTAGCA
GmINV3	GATGGTCAGGAGTGGCAGAT	GGTAGGACCAAGGCGTGTT

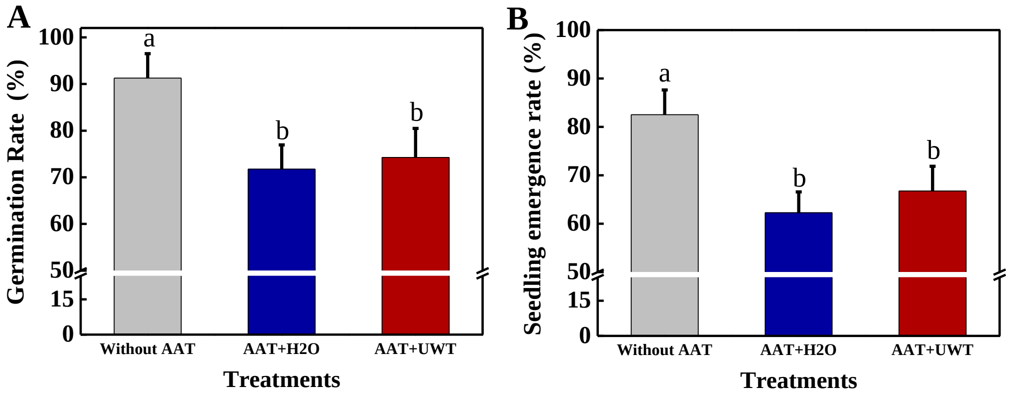

Fig.S1. UWT made no effect on germination (A) and seedling emergence (B) of artificially-aged soybean seeds

Without AAT: fresh seeds without accelerated aging test (AAT); AAT + H_2O: AAT seeds with H_2O; AAT + UWT: AAT seeds with ultrasonic waves treatment (UWT). The diverse lowercase(s) on top of the bars were indicative of significant differences ($p<0.01$, Lsd) across treatments.
